# Human cancer stem cells are a target for cancer prevention using (−)-epigallocatechin gallate

**DOI:** 10.1007/s00432-017-2515-2

**Published:** 2017-09-23

**Authors:** Hirota Fujiki, Eisaburo Sueoka, Anchalee Rawangkan, Masami Suganuma

**Affiliations:** 1 Faculty of Medicine, Saga University, Nabeshima, Saga, 849-8501 Japan; 2 Graduate School of Science and Engineering, Saitama University, Saitama, 338-8570 Japan

**Keywords:** CSCs, EGCG, Parental cells, Self-renewal, Stemness

## Abstract

**Purpose:**

Our previous experiments show that the main constituent of green-tea catechins, (−)-epigallocatechin gallate (EGCG), completely prevents tumor promotion on mouse skin initiated with 7,12-dimethylbenz(a)anthracene followed by okadaic acid and that EGCG and green tea extract prevent cancer development in a wide range of target organs in rodents. Therefore, we focused our attention on human cancer stem cells (CSCs) as targets of cancer prevention and treatment with EGCG.

**Methods:**

The numerous reports concerning anticancer activity of EGCG against human CSCs enriched from cancer cell lines were gathered from a search of PubMed, and we hope our review of the literatures will provide a broad selection for the effects of EGCG on various human CSCs.

**Results:**

Based on our theoretical study, we discuss the findings as follows: (1) Compared with the parental cells, human CSCs express increased levels of the stemness markers Nanog, Oct4, Sox2, CD44, CD133, as well as the EMT markers, Twist, Snail, vimentin, and also aldehyde dehydrogenase. They showed decreased levels of E-cadherin and cyclin D1. (2) EGCG inhibits the transcription and translation of genes encoding stemness markers, indicating that EGCG generally inhibits the self-renewal of CSCs. (3) EGCG inhibits the expression of the epithelial-mesenchymal transition phenotypes of human CSCs. (4) The inhibition of EGCG of the stemness of CSCs was weaker compared with parental cells. (5) The weak inhibitory activity of EGCG increased synergistically in combination with anticancer drugs.

**Conclusions:**

Green tea prevents human cancer, and the combination of EGCG and anticancer drugs confers cancer treatment with tissue-agnostic efficacy.

## Introduction

Cancers develop from a small subset of cancer stem cells (CSCs). A CSC, as defined by attendees of an American Association for Cancer Research Workshop on Cancer Stem Cells (Clarke et al. 2006), is “a cell within a tumor that possesses the capacity to self-renew and to cause the heterogeneous lineages of cancer cells that comprise the tumor”. In a simple model, cells of the epidermis are classified into two types of proliferating cells as follows: stem cells with high proliferative potential (self-renewal) and transit amplifying cells (progenitor cells) with a low capacity for self-renewal and a high probability of undergoing terminal differentiation (Jones and Watt 1993). The role of human cancer stem cells (CSCs) is now a topic of cancer prevention, and inhibitors of CSCs are attracting much attention as potential therapeutics (Yoshida and Saya 2016).

We found that repeated applications of the green-tea component 5 mg (−)-epigallocatechin gallate (EGCG) before each treatment with 1 μg okadaic acid, a potent tumor promoter and inhibitor of protein phosphatases 1 and 2A (Fujiki and Suganuma 1993), completely prevents tumor promotion on mouse skin in two-stage carcinogenesis experiments initiated using 7,12-dimethylbenz(a)anthracene (DMBA). In contrast, tumors formed in 73.3% of mice treated with DMBA plus okadaic acid at week 20 (Yoshizawa et al. 1992; Fujiki and Okuda 1992).

Breast cancer patients who consume >5 cups of green tea per day (average eight cups) experience lower recurrence rated compared with patients who consume <4 cups daily (average three cups) (Nakachi et al. 1998). Further, drinking 10 Japanese-size cups (120 ml/cup) of green tea per day delays cancer onset by 7.3 years in female patients and significantly prevents cancers of the lungs, colorectum, liver, and stomach in patients residing in Saitama Prefecture, Japan (Imai et al. 1997; Nakachi et al. 2000). The prevention of the development of prostate cancer in patients with high-grade prostate intraepithelial neoplasia using capsules of green tea catechins was confirmed in a study conducted in Italy (Bettuzzi et al. 2006). A double-blind randomized clinical phase II prevention trial conducted at Gifu University, Japan revealed that drinking 10 Japanese-size cups of green tea supplemented with green tea tablets prevents the recurrence of 51.6% of colorectal adenomas (Shimizu et al. 2008).

A similar trial conducted at Seoul National University found that laboratory-made tablets of green tea extract and placebo showed 44.2% prevention rate for the recurrence of colorectal adenoma (Shin et al. 2017), because of the reduction of the tumor rate between the groups treated with green tea extract and placebo. Moreover, oral premalignant leukoplakia was prevented in patients at the University of Texas M. D. Anderson Cancer Center who were treated with green tea extract (Tsao et al. 2009). Further, an encouraging development in the field was the design of the first large-scale placebo-controlled trail for the prevention of metachronous adenoma recurrence in the colorectum of patients to be administered green tea extract for 3 years at the University Ulm, Germany (Stingl et al. 2011). The potential significant preventative effects of EGCG are extended to an innovative treatment. Thus, the combination of EGCG or green tea extract and anticancer compounds such as retinoids, nonsteroidal anti-inflammatory drugs (NSAID), paclitaxel, and doxorubicin synergistically enhances the anticancer activity in human cancer cell lines and in mouse xenograft models (Suganuma et al. 2006, 2011; Fujiki et al. 2015; Stearns and Wang 2011).

^3^H-EGCG binds to the cell surface membrane and is incorporated into the cytosol and nucleus (Okabe et al. 1997). One to three EGCG molecules bind to single-stranded 18-mers of DNA and RNA (Kuzuhara et al. 2006). Moreover, EGCG inhibits the formation of primary and secondary spheroids and the expression of the pluripotency-maintaining factor genes *Nanog*, *c*-*Myc*, and *Oct4* in human prostate and pancreatic CSCs (Tang et al. 2010, 2012). A genetic labeling strategy revealed CSCs during the unperturbed growth of solid tumors on mouse skin (Driessens et al. 2012). These results encouraged us to study whether self-renewal of human CSCs can be prevented using EGCG and whether the combination of EGCG and anticancer drugs reduces the stemness of human CSCs.

We conducted a PubMed search that identified papers reporting the anticancer activity of EGCG against 20 different kinds of human CSCs. For example, the self-renewal of cancer stem-like cells enriched using a spheroid formation assay was investigated (Sugihara and Saya 2013). Numerous investigators identified proteins expressed by CSCs and used them as markers to investigate the inhibitory effects of EGCG on stemness (self-renewal and differentiation). Since similar effects of EGCG on CSCs of various human cancer cell lines have been confirmed, this review of the literature will be useful for the readers to check all collected results, as the whole, and to demonstrate that EGCG inhibits the essential mechanisms that maintain CSCs, because EGCG reduces the expression of stemness markers.

This review discusses the important topics as follows: (1) Human CSCs in four different cancer tissues differentially express stemness markers compared with their respective parental cells. (2) EGCG inhibits the expression by human CSCs of stemness markers such as transcription factors and other proteins that contribute to generating epithelial-mesenchymal transition (EMT) phenotypes, indicating that EGCG generally inhibits the self-renewal of CSCs in cancer tissues. (3) EGCG inhibits the expression of EMT phenotypes by CSCs. (4) The inhibitory effects of EGCG on the stemness of CSCs are weaker compared with those on parental cells. (5) The weak inhibitory effects on the proliferation and differentiation of CSCs by EGCG are synergistically increased using EGCG combined with anticancer drugs. Thus, the combination inhibits the viability of human CSCs.

## Human CSCs differentially express stemness markers compared with their parental cells

Human CSCs enriched from primary and secondary spheroids are capable of self-renewal. It is important to study the quantitative differences between the levels of stemness markers between CSCs and parental cells, because CSCs redifferentiate into parental cells when cultured in parental growth conditions, and parental cell lines do not form tumorspheres (Dalla Pozza et al. 2015).

This section reviews studies of six human CSCs and their respective parental cells, which were derived from colorectal cancer (CRC), nasopharyngeal cancer, neuroblastoma, and glioblastoma (Table 1). The human CRC, spheroid-derived CSCs, designated HCT116-SDCSCs, express significantly higher levels of stem cell markers (approximately 4.5-fold and 3.2-fold for *Oct4* and *Nanog*, respectively, compared with the parental cells) (Toden et al. 2016). Oct, Nanog, and Sox2 are transcription factors required for the maintenance of pluripotency by coordinated networks of transcription factors (Kashyap et al. 2009; Sarkar and Hochedlinger 2013; Boumahdi et al. 2014). Moreover, the expression levels of the surface marker CD44 and self-renewal markers Notch, Bmi-1, CD133, and ALDH1 are higher in HCT116-SDCSCs compared with parental cells (Table 1). Aldehyde dehydrogenase (ALDH) is a detoxifying enzyme that catalyzes the oxidation of retinal to retinoic acid (Duester 2000) and is used to isolate stem-like breast cancer cells, which are characterized by their enhanced tumorigenicity and ability to self-renew (Ginestier et al. 2007). The results indicate that HCT116-SDCSCs comprise a higher CSC population compared with that of the parental cell population (Toden et al. 2016).

**Table 1:** Human CSCs differentially express stemness markers

Cancers and names of CSCs	Markers of increased expression	Markers of decreased expression	References
Colorectal cancer			
HCT116-SDCSCs	mRNAs: *Oct4*, *Nanog*,		Toden et al. (2016)
	Proteins: CD44, Notch, Bmi-1, CD133, ALDH1		
Nasopharyngeal cancer			
TW01 sphere	mRNAs: *Sox2, Oct4, KLf*-*4*, *Twist, Snail, vimentin, N*-*cadherin*	*E*-*cadherin*	Lin et al. (2012)
CNE2- & C666-1-SCs	mRNAs: *CD44, Bmi*-*1*, *N*-*cadherin, vimentin*, *Twist*	*E*-*cadherin*	Li et al. (2015)
	Proteins: CD44		
Neuroblastoma			
BE(2)-C sphere	mRNAs: *Nanog, Oct4,*	*ATP7A, DKK2*	Nishimura et al. (2012)
Glioblastoma			
U87 GSLCs	mRNAs: *ALDH1, CD133,*	*GFAP*	Zhang et al. (2015)
	Proteins: CD133, ALDH1,	Cyclin D1	

Human nasopharyngeal sphere-derived cells CSCs, designated TW01, express relatively high levels of the stem cell markers Sox2, Oct4, and KLf-4, plus EMT markers including Twist, Snail, and vimentin, along with N-cadherin, compared with parental cells. The relative increases of mRNA levels decreases in the order *vimentin*, *Snail*, *Oct*4, *Sox2*, *Twist*, *KLf*-*4,* and *N*-*cadherin* (Table 1). However, decreased expression of *E*-*cadherin* by TW01 sphere-derived cells was detected (Lin et al. 2012). The results indicate that TW01 sphere-derived cells express EMT phenotypes and self-renewal activity associated with invasive and migratory properties more strongly compared with those of parental cells. Similarly, the human nasopharyngeal spheroid cell lines CNE2-SCs and C666-1-SCs express increased levels of the mRNAs of the stemness markers *CD44* and *Bim*-*1*, the mesenchymal markers *N*-*cadherin* and *vimentin*, and the transcription factor *Twist* compared with those of their respective parental cells (Table 1). Further, decreased levels of the epithelial marker *E*-*cadherin* are expressed by CNE2-SCs and C666-1-SCs. The expression of the CSC marker CD44 by CNE2-SCs and C666-1-SCs is significantly increased compared with that of their respective parental cells (Li et al. 2015). CD44 is an adhesion molecule that binds to osteopontin and hyaluronic acid (Goodison et al. 1999), and CD44^+^ cells in a tumor express high levels of nuclear Bim-1, which identifies CSCs (Prince et al. 2007). These results show that CNE2-SCs and C666-1-SCs have a stronger ability to self-renew and migrate compared with their respective parental cells, suggesting that the tumor spheroid cells are enriched with malignant phenotypes (Li et al. 2015).

Human neuroblastoma BE(2)-C spheres express increased levels of *Nanog* and *Oct4* (approximately 50-fold and 5.0-fold, respectively) compared with the parental cells (Nishimura et al. 2012), consistent with findings for human HCT116-SDCSCs (Toden et al. 2016). The expression levels of markers of neuronal differentiation, Cu^2+^-transporting ATPase alpha polypeptide (*ATP7A*) and dickkopf homolog 2 (*DKK2*), are slightly decreased in BE(2)-C spheres (Table 1). These results indicate that BE(2)-C spheres are enriched in tumor-initiating cells as well as CSCs that are responsible for resistance to chemotherapy and tumor recurrence (Nishimura et al. 2012).

Human glioma stem-like cells designated U87 GSLCs express increased levels of the mRNAs and proteins of the stem cell markers *ALDH1* and *CD133*, decreased levels of the astrocyte differentiation marker glial fibrillary acidic protein (*GFAP*) (Pang et al. 2017) and cell cycle protein cyclin D1, compared with the parental cells U87 (Table 1) (Zhang et al. 2015). ALDH1 is a marker of normal and malignant human mammary stem cells (Ginestier et al. 2007), and GFAP is an intermediate filament protein that is expressed by numerous cell types of the central nervous system, but may represent an autoantigen candidate for type 1 diabetes mellitus (Pang et al. 2017). These results indicate that U87 GSLCs comprise neural stem cells with drug-resistant properties (Zhang et al. 2015).

## EGCG inhibits the expression of mRNAs and proteins that serve as stemness markers of human CSCs

EGCG and green tea extract inhibit the growth of human cancer cell lines in culture and in rodents (Suganuma et al. 1998; Fujiki et al. 2002, 2012). Therefore, we next studied whether EGCG possesses anticancer activity against drug-resistant human CSCs. The results of studies on 20 human CSCs collected from nine different cancer tissues reveal that EGCG inhibits the expression of mRNAs and proteins that serve as stemness markers, such as *Oct4* and *Nanog*, whereas the expression of a few markers such as Bax and caspase 8 is increased, indicating that EGCG exerts inhibitory effects on human CSCs (Table 2).

**Table 2:** EGCG decreases or increases the expression of stemness marker mRNAs and proteins by human CSCs

Cancers and names of CSCs	Inhibited expression of stemness markers (mRNAs and proteins)	References
Breast CSCs		
SUM-149 & SUM-190	mRNAs: *CCND1, RHOC, BCL*-*XL*	Mineva et al. (2013)
SUM-149	mRNAs: *FN1, CDHI, vimentin*	
MDA-MB-231 & MDA-MB-436	Proteins: ER-α36, EGFR, p-ERK1/2, p-AKT	Pan et al. (2016)
Lung CSCs		
A549 & H1299	mRNAs: *CD133, CD44, ALDH1A1, Nanog, Oct4*	Zhu et al. (2017)
	Proteins: CD133, CD44, ALDH1A1, Nanog, Oct4, PCNA, cyclin D1, Bcl2, p-GSK3β, β-catenin, c-Myc	
	Increased Bax, caspase 8, cleaved caspase-3 and -9	
Prostate CSCs		
PC-3 & LNCaP	Proteins: XIAP, Bcl2, survivin, vimentin, Slug, Snail, β-catenin	Tang et al. (2010)
Colorectal CSCs		
HCT116-5FUR & SW480-5FUR	mRNAs: *Oct4, Nanog*	Toden et al. (2016)
	Proteins: Notch 1, cleaved-Notch 1, c-Myc, Bmi-1, Suz12, Ezh2	
HCT116-SDCSCs	mRNAs: *CD133, Nanog, ABCC1, ABCG2*	Wubetu et al. (2016)
	Proteins: Nek2, p-Akt	
Liver CSCs		
HepG2-SDCSCs	mRNAs: *CD133, Nanog, ABCC1, ABCG2,*	Wubetu et al. (2016)
	Proteins: Nek2, p-Akt	
Pancreatic CSCs		
Pancreatic CSCs	mRNAs: *Nanog, c*-*Myc, Oct4*, *Bcl2, survivin, XIAP*, *SMO, PTCH1, PTCH2, Gli1, Gli2*, *Snail, ZEB1, Slug*	Tang et al. (2012)
MIA-PaCa2 & BxPc-3	mRNA: *K*-*ras* (by green tea extract)	Appari et al. (2014)
Head and neck CSCs		
HNSC (K3, K4, K5)	mRNAs: *Oct4, Sox2, Nanog, Notch1, Hey1, Hes1 ABCC2, ABCG2*	Lee et al. (2013)
	Proteins: Oct4, Sox2, Notch1, Hey1, Hes1, ABCC2, ABCG2	
	Increased *involucrin*	
Nasopharyngeal CSCs		
TW01 & TW06 spheres	mRNAs: *Oct4, KLf*-*4*, *vimentin, Snail* Increased *E*-*cadherin*	Lin et al. (2012)
Glioma CSCs		
U87 GSLCs	mRNAs: *ABCB1, ABCG2, MGMT*	Zhang et al. (2015)
	Proteins: p-Akt, CD133, ALDH1, Bcl2, P-gp	
	Increased Bax, c-PARP	

Breast CSCs: EGCG (40 μg/ml, 87.3 μM) inhibits the expression of genes that promote growth and contribute to the transformed phenotype and survival of SUM-190 spheres. In SUM-149 and SUM-190 cells, EGCG decreases the levels of mRNAs of the proliferation markers cyclin D1 (*CCND1)*; ras homolog family member C (*RHOC*); and B-cell lymphoma-extra large (*BCL*-*XL*), which is a major antiapoptotic protein of the Bcl2 family, as well as decreasing ATP levels. In contrast, the levels of fibronectin 1 (*FN1*), E-cadherin (*CDHI*), and *vimentin* were decreased only in SUM-149 cells (Table 2). These results indicate that tumorsphere formation was inhibited by EGCG (Mineva et al. 2013).

ALDH-positive SUM-149 cells that are functionally active CSCs were implanted into the inguinal mammary fat pad of female non-obese mice with diabetic/severe-combined immunodeficiency. When palpable tumors were detected, mice were administered a 0.1 ml intraperitoneal injection of 16.5 mg/kg EGCG or control PBS five times weekly for 5 weeks. Tumor weight significantly decreases by 28.6 ± 6.5% in EGCG-treated mice (Mineva et al. 2013). The phenotypes of human estrogen receptor (ER)-negative MDA-MB-231 and MDA-MB-436 cells reflect tumors with a poor prognosis. In ER-negative breast cancer cell lines, ER-α36 is overexpressed and is associated with malignant growth (Zhang et al. 2011). EGCG (10–40 μM) inhibits tumorsphere formation and down-regulates ER-α36 expression by 24 h, which is consistent with down-regulation of the epidermal growth factor receptor (EGFR). We found it interesting that the inhibitory effects of EGCG are weak in ER-α36 knockdown cell lines compared with control cells. However, EGCG inhibits the growth of ER-negative human breast CSCs through down-regulation of ER-α36 expression, indicating that EGCG treatment will predict longer survival of patients with mammary cancers (Pan et al. 2016) (Table 2). The longer survival of patients with green tea was reported by Nakachi et al. (1998), as noted in the Introduction.

Lung CSCs: EGCG (0–100 μM) reduce the mRNA levels of the lung CSC markers *CD133*, *CD44*, *ALDH1A1*, *Nanog,* and *Oct4* in CSC-A549 and CSC-H1299 cells; the protein levels of proliferation markers proliferating cell nuclear antigen (PCNA) and cyclin D1 as well as that of B-cell lymphoma 2 (Bcl2). In contrast, EGCG reduces the protein levels of β-catenin and c-Myc. However, EGCG increases the levels of Bax, caspase 8, and cleaved caspases-3 and -9 (Table 2). These results show that EGCG inhibits proliferation and induces apoptosis of lung CSCs (Zhu et al. 2017). The Wnt/β-catenin pathway is implicated in self-renewal and maintenance of CSCs, and it is dysregulated in human cancers (Jang et al. 2015). EGCG (0–100 μM) inhibited the phosphorylation of glycogen synthase kinase 3β (GSK3β) at Ser 9, which significantly increases the expression of GSK3β, and decreases the expression of β-catenin and its downstream target gene *c*-*Myc*. Thus, EGCG down-regulates the activation of the Wnt/β-catenin pathway in human lung CSCs (Zhu et al. 2017; Oh et al. 2014) (Table 2).

Prostate CSCs: EGCG (0–60 μM) dose-dependently inhibits the growth of tumor spheroids and the self-renewal of prostate CSCs derived from PC-3 and LNCaP cell lines. Nanog is a key regulator of embryonic stem cell self-renewal and pluripotency (Jeter et al. 2009), and the knockdown of Nanog by a short hairpin RNA enhances the antiproliferative effects of EGCG on prostate CSCs (Tang et al. 2010). EGCG (30–60 μM) inhibits the expression of X-linked inhibitor of apoptosis protein (XIAP), Bcl2, and survivin as well as that of the EMT markers vimentin, Slug, Snail, and nuclear β-catenin. These results indicate that EGCG will serve as a useful compound that targets prostate CSCs (Tang et al. 2010) (Table 2).

Colorectal CSCs: 5-Fluorouracil (5FU)-resistant (5FUR) CRC cells exhibit an increased ability to form spheroids compared with parental cells, indicating the presence of a larger CSC population. EGCG (50 μM) inhibits tumorspheroid formation and the expression of the mRNAs of the stem cell markers *Oct4* and *Nanog*. The expression of self-renewal markers that are components of the Notch signaling pathway, Notch1, cleaved-Notch1, and c-Myc as well as that of the polycomb repressive complex subunits Bmi-1, polycomb protein SUZ (Suz12), and enhancer of zeste homologue (Ezh2) is inhibited in 5FUR CRCs treated with EGCG (Toden et al. 2016). EGCG (50 μM) down-regulates the expression of the ATP-binding cassette transporter genes ATP-binding cassette subfamily C member 1 (*ABCC1*) and *ABCG2* and decreases the protein levels of never in mitosis gene related kinase 2 (Nek2), which is involved in mitotic regulation, and phosphorylated Akt, a proto-oncoprotein, in HCT116-SDCSCs (Wubetu et al. 2016).

Liver CSCs: EGCG (50 μM) down-regulates the expression of *CD133, Nanog, ABCC1, and ABCG2* mRNAs as well as that of the Nek2 and p-Akt proteins in human hepatoma HepG2-CSCs to an extent similar to that of HCT116-SDCSCs (Wubetu et al. 2016).

Pancreatic CSCs: Human pancreatic CSCs (CD133^+^/CD44^+^/CD24^+^/epidermal surface antigen ESA^+^) represent the bulk of the pancreatic CSC population. EGCG (0–60 μM) inhibits the growth of tumorspheroids. EGCG inhibits the expression by human pancreatic CSCs of the mRNAs of the pluripotency maintaining transcription factors *Nanog*, *c*-*Myc,* and *Oct4*; the apoptosis-related proteins *Bcl2*, *survivin*, and *X*-*linked inhibitor of apoptosis protein* (*XIAP*); the Sonic hedgehog (Shh) receptors *smoothened* (*SMO*), *Patched*-*1* (*PTCH1*), and *Patched*-*2* (*PTCH2*); the transcription factors *Gli1* and *Gli2*; and the EMT factors *Snail*, *ZEB1*, and *Slug* (Table 2). In contrast, EGCG does not inhibit the expression of *Sox2* (Tang et al. 2012).

Numerous Sox factors act redundantly in the maintenance of stem cells (Sarkar and Hochedlinger 2013). The inhibition of *Nanog* expression by EGCG is important for cancer prevention. The human pancreatic ductal adenocarcinoma (PDA) cell line MIA-PaCa2 has characteristic CSC features. EGCG (40 μM), (−)-epicatechin gallate (ECG, 40 μM), or (−)-catechin gallate (CG, 40 μM) decrease colony-forming activity, and ECG or CG exert stronger effects compared with those of EGCG. Treatment of Mia-PaCa2, BxPc-3, and CSC-enriched PDA cells (PacaDD-183) combined with 40 μM green tea extract enhances the expression of the tumor suppressor microRNA (*miR*-*let*-*7a*), which suppresses the expression of genes encoding stemness markers by binding to the 3′-untranslated region of target mRNAs (Yu et al. 2008, 2016). Further, miR-let-7a inhibits the expression of K-ras (Appari et al. 2014). These results indicate that green-tea catechins inhibit colony formation.

Head and neck CSCs: Treatment of three head and neck squamous carcinoma (HNSC) CSCs (K3, K4, and K5) with EGCG (5 and 10 μM) significantly inhibit sphere formation, suggesting that EGCG effectively inhibits the self-renewal of HNSC CSCs. EGCG inhibits the expression of the stem cell markers *Oct4*, *Sox2*, and *Nanog* and increases the expression of the early differentiation marker *involucrin*. EGCG decreases the expression of the mRNAs of the ABC transporters *ABCC2* and *ABCG2* and ABCC2 and ABCG2 proteins, suggesting that suppressing the transcription of *ABCC2* and *ABCG2* transporter genes with EGCG augments cisplatin-mediated chemosensitivity. Further, EGCG inhibits the expression of mRNAs of Notch signaling-pathway target genes *Notch1*, *Hey1*, and *Hes1* and the expression of the proteins Notch1, Hey1, and Hes1 by K3 CSCs (Lee et al. 2013). In contrast, the Notch pathway ligands Delta1, Jag 1, and Jag2 are not affected in cochlear explant culture by EGCG (Gu et al. 2015).

Nasopharyngeal CSCs: EGCG (20–40 μM) inhibits colony formation by TW01 and TW06 sphere-derived cells. EGCG inhibits the expression of the mRNAs of the stemness markers *Oct4* and Krüppel-like factor (*KLf*-*4*) but not that of the mRNAs of *Sox2* and the EMT related proteins vimentin and Snail, but increases that of *E*-*cadherin* in TW01 sphere-derived cells (Lin et al. 2012). KLf-8 plays a key role in oncogenic transformation, induces the EMT, and enhances motility and invasiveness (Wang et al. 2007).

Glioma CSCs: EGCG (0–200 μM) inhibits cell viability, neurosphere formation, and the migration of human glioma stem-like cells (U87 GSLCs). Although EGCG inhibits the expression of the mRNAs of the proteins that confer drug resistance, such as P-glycoprotein (*P*-*gp, ABCB1*), *ABCG2*, and *O*-6-methylguanine-DNA methyltransferase (*MGMT*) in U87 GSLCs, it does not change the protein level of ABCG2. Further, EGCG down-regulates p-Akt as well as the levels of the glioma stem cell markers CD133, ALDH1, Bcl2, and P-gp. In contrast, EGCG increases the levels of Bax and cleaved poly-ADP-ribose polymerase (c-PARP) proteins in U87 GSLCs (Zhang et al. 2015).

## EGCG inhibits the expression of EMT phenotypes of CSCs

Tumor progression is frequently associated with up-regulation of vimentin and transcription factors such as Snail, Twist, and Slug as well as down-regulation of E-cadherin (Polyak and Weinberg 2009; Thiery et al. 2009). Functional loss of the cell–cell adhesion molecule E-cadherin is required for the EMT, which induces migration, invasion, and stem cell features (Sobrado et al. 2009). E-cadherin repressors are classified into two groups depending on their effects on the *E*-*cadherin* promoter as follows: Inducers of the EMT and repressors of E-cadherin, such as Snail, zinc finger E-box-binding homeobox (Zeb), E2A immunoglobulin enhancer-binding factor (E47), and KLf-8 that binds to and represses the activity of the *E*-*cadherin* promoter (Peinado et al. 2007; Wang et al. 2007). In contrast, Twist, homeobox protein goosecoid (Goosecoid), basic helix-loop-helix protein (E2.2), and Forkhead box C1 (FoxC1) indirectly repress *E*-*cadherin* transcription (Yang and Weinberg 2008).

We found that EGCG (50 μM) inhibits high expression of vimentin and Slug in H1299 human non-small cell lung cancer cells at a leading edge of a scratch and that EGCG increases the stiffness (Young’s modulus) of H1299 and Lu99 cells, suggesting that EGCG inhibits EMT phenotypes through the alterations of membrane organization (Takahashi et al. 2014; Suganuma et al. 2016). The combination of EGCG and cisplatin markedly down-regulate the expression of NF-κB p65, Bmi-1, Twist1, and vimentin and up-regulates E-cadherin in human nasopharyngeal CNE2-SCs from the tumor nodules of nude mice, which is associated with the inhibition of tumorigenesis (Li et al. 2015). We found that EGCG in drinking water inhibits the formation of hematogenous and lymphogenous (spontaneous) metastases by two different B16 melanoma variants in the lungs of C56BL/6 mice (Taniguchi et al. 1992). EGCG induces a twofold increase in the stiffness of highly motile B16-F10 melanoma cells and inhibits their migration (Watanabe et al. 2012).

## Inhibitory effects of EGCG on CSCs are weaker than on parental cells

**Fig. 1:**
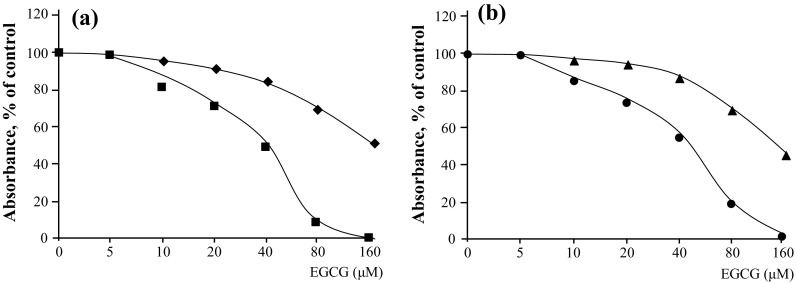
EGCG inhibits the proliferation of nasopharyngeal CSCs and parental cells. MTT assays of (**a**) TW01 sphere-derived cells (*square*) and TW01 parental cells (*filled square*) and (**b**) TW06 sphere-derived cells (*filled triangle*) and TW06 parental cells (*filled circle*) (Lin et al. 2012, Reproduced with the permission of Dr. Yann-Jang Chen)

The acquisition of drug resistance by cancer cells presents a formidable clinical challenge, particularly because drug resistance enables tumor recurrence. When EGCG is administered for cancer prevention, it is necessary to consider the different potencies of EGCG against CSCs and parental cells. Two investigations of nasopharyngeal CSCs and neuroblastoma CSCs found that the effects of EGCG on the inhibition of cell proliferation and the induction of apoptosis differed slightly between sphere-derived and parental cells (Table 3). Specifically, the ID_50_ (dose ″μM″ required to achieve 50% inhibition) of EGCG was calculated from the curves of MTT assays (Fig. 1a, b). The ID_50_ values of EGCG treatment of TW01 sphere-derived cells and parental cells are approximately 150 and 60 μM, respectively, and approximately 115 μM EGCG and 38 μM EGCG for TW06 sphere-derived and parental cells, respectively (Fig. 1a, b; Table 3) (Lin et al. 2012). Treatment of BE(2)-C sphere and BE(2)-C parental cells with 50 μM EGCG inhibits cell proliferation by 25.2 and 93.8%, respectively, compared with non-treated cells (Nishimura et al. 2012). Induction of apoptosis using EGCG was determined in sphere-derived and parental TW01 and BE(2)-C cells (Table 3). The results indicate that EGCG is a less effective inhibitor of the sphere-derived cells compared with parental cells, although EGCG inhibits the growth of both.

**Table 3:** Inhibitory effects of EGCG on CSCs are weaker than those on parental cells

(A) Inhibition of cell proliferation using EGCG			
**Nasopharyngeal cancer**	TW01 sphere	TW01 parental	Lin et al. (2012)
ID_50_ μM	150	60	
	TW06 sphere	TW06 parental	Lin et al. (2012)
ID_50_ μM	115	38	
**Neuroblastoma**	BE(2)-C sphere	BE(2)-C parental	Nishimura et al. (2012)
EGCG 50 μM	25.2 (% of inhibition)	93.8 (%)	
(B) Induction of apoptosis using EGCG			
**Nasopharyngeal cancer**	TW01 sphere	TW01 parental	Lin et al. (2012)
EGCG 40 μM	12.7 (%)	63.6 (%)	
**Neuroblastoma**	BE(2)-C sphere	BE(2)-C parental	Nishimura et al. (2012)
EGCG 50 μM	9.1 (%)	91.7 (%)

## EGCG combined with anticancer drugs synergistically enhances anticancer activity against human CSCs

We found that the combination of green tea extract and NSAIDs synergistically inhibits tumor development in rodents and induces apoptosis associated with expression of the growth arrest and DNA damage-inducible gene 153 (*GADD153*) and *p21* in the human lung cancer cell line PC-9 (Suganuma et al. 1999, 2011). Subsequently, numerous investigators extended these studies by combining EGCG with other green-tea catechins and anticancer drugs in 42 in vitro studies and 13 studies of mouse xenograft models (Fujiki et al. 2015). The combination of EGCG with anticancer drugs frequently induced synergistic anticancer activity against human cancer cell lines.

Considering the evidence indicating that the effects of EGCG on human CSCs are slightly weaker compared with the parental cells, a new strategy is required to increase efficacy. We conducted a literature search that identified studies of five human CSCs treated with the combinations (Table 4). Docetaxel inhibits mitosis, and the combination of EGCG, quercetin, and docetaxel reduces colony formation by two prostate CSC lines compared with controls (Wang et al. 2015) (Table 4). 5FU damages cancer cells during S phase, and cisplatin damages DNA, leading to the induction of apoptosis (Fujiki et al. 2015). The combination of EGCG and 5FU or EGCG and cisplatin reduces the weights of tumors formed by three CSCs derived from the colorectum, head and neck, and nasopharynx (Toden et al. 2016; Lee et al. 2013; Li et al. 2015). The combination of EGCG and anticancer drugs is a promising therapeutic strategy for treating human CSCs.

**Table 4:**
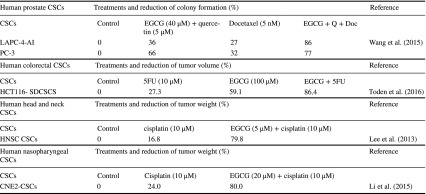
Combinations of EGCG and anticancer drugs synergistically enhance anticancer activity against human CSCs

## Discussion

It is necessary to briefly discuss the effects of EGCG on normal cells. The antiangiogenic effects of EGCG are not observed in normal endothelial cells (NECs), compared with tumor-associated endothelial cells (TECs) and endothelial progenitor cells (EPCs). The reason why EGCG is inactive on NECs is explained by evidence indicating that the stem cell marker stem cell antigen-1 (*Sca*-*1*) is expressed at a low level in NECs, although it is overexpressed by TECs and EPCs (Ohga et al. 2009). Sca-1 was originally identified as an 18-kDa glycoprotein 1-binding surface protein and is a member of the lymphocyte activation protein-6 family.

The difference between cancer cells and normal cells is a vital subject: Treatment with 87.3 μM EGCG for 48 h resulted in apoptosis in human epidermoid carcinoma cell line (A431), human carcinoma keratinocyte (HaCaT), and human prostate carcinoma cell line (DU145), but 349.1 μM EGCG was not effective on normal human epidermal keratinocytes (NHEK) (Ahmad et al. 1997). EGCG (10–80 μM) for 24 h results in lowering of NF-κB levels in both the cytoplasm and nucleus in a dose-dependent manner in both A431 and NHEK cells. However, EGCG-mediated inhibition of NF-κB constitutive expression and activation, only occurred at a higher dose of EGCG (40–80 μM) in NHEK, compared with A431 cells (Ahmad et al. 2000).

As reported in Introduction, a single topical application of 5 mg was applied to mouse skin. The specific binding of ^3^H-TPA and that of ^3^H-okadaic acid to the receptors in a membrane fraction of normal mouse skin were then determined. Both specific bindings decreased immediately, reaching minimum in 5–10 min. The levels gradually returned to normal, suggesting that EGCG induces the sealing of the membrane on mouse skin because of EGCG-protein interaction (The sealing effects of EGCG), resulting in inhibition of tumor promotion and tumor development associated with internalization of ^3^H-EGCG (Yoshizawa et al. 1992; Okabe et al. 1997).

In contrast to our initial work, recent investigation on the interaction of EGCG and cellular molecules, along with receptor activation and gene expression, have shown outstanding progress in the elucidation of therapeutic mechanisms for green tea catechin: Excessive concentrations of vascular endothelial growth factor (VEGF) increase growth of tumors. VEGF stimulates cellar responses by binding to VEGF receptor-2 (type III receptor tyrosine kinases) on the cell surface, resulting in phosphorylation of VEGFR-2. Treatment of human umbilical vein endothelial cells (HUVECs) with 1 μM EGCG resulted in complete inhibition of VEGF-induced VEGFR-2 activation, indicating that direct interaction of EGCG with VEGF is a significant mechanism. In these circumstances, endothelial nitric oxide synthase is still activated through the PI3K/Akt signaling pathway that is downstream of VEGFR-2 (Moyle et al. 2015). Matrix metalloproteinases (MMPs) are involved in tumor progression, and treatment with 10.9–87.3 μM EGCG inhibited fibroblast conditioned medium (FCM)-induced phosphorylation of ERK1/2 and/or p38 concomitant reduction in MMP-2 and -9 in human prostate carcinoma DU-145 cells. It also inhibited androgen-induced pro-MMP-2 expression in androgen-sensitive human prostate adenocarcinoma LNCaP cells (Vayalil and Katiyar 2004).

## Summary

EGCG and green tea extracts effectively prevent the early stages of cancer, and the combination of EGCG and anticancer drugs is more effective for inhibiting the viability of human CSCs.

## Abbreviations

ABC: ATP-binding cassette
AFM: Atomic force microscopy
ALDH: Aldehyde dehydrogenase
CSCs: Cancer stem cells
CRC: Colorectal cancer
DMBA: 7,12-Dimethylbenz(a)anthracene
EGCG: (−)-Epigallocatechin gallate
EGFR: Epidermal growth factor receptor
EMT: Epithelial-mesenchymal transition
ER: Estrogen receptor

## Glossary

ABCB1: ATP-binding cassette subfamily B member 1
ABCG2: ATP-binding cassette subfamily G member 2
Akt: RAC-alpha serine/threonine-protein kinase
Bcl2: B-cell lymphoma 2
Bax: Bcl-2-associated X protein
Bmi-1: B-lymphoma Moloney murine leukemia virus integration site 1 homologue
ERK1/2: Extracellular-signal regulated kinase 1/2
GP1: Glycoprotein 1
Gli1: Zinc finger protein GLl1
Hey1: Hairy/enhancer-of-split related with YRPW motif protein 1
K-ras: KRAS oncogene
KLf: Krüppel-like factor
Myc: v-Myc avian myelocytomatosis viral oncogene homolog
Nanog: Nanog homeobox protein
Notch1: Notch homolog 1
Oct4: Octamer-binding transcription factor 4
Patched-1: Protein patched homolog 1
SMO: A class Frizzled (class F) G protein-coupled receptor
Slug: SNAI2
Snail: Snail family transcriptional repressor
Sox2: SRY (sex determining region Y)-box 2
Twist: Twist family BHLH transcription factor
Wnt: Mammalian homologs of *Drosophila* ‘wingless” signaling

## References

[CR1] Ahmad N, Feyes DK, Nieminen AL, Agarwal R, Mukhtar H (1997). Green tea constituent epigallocatechin-3-gallate and induction of apoptosis and cell cycle arrest in human carcinoma cells. J Natl Cancer Inst.

[CR2] Ahmad N, Gupta S, Mukhtar H (2000). Green tea polyphenol epigallocatechin-3-gallate differentially modulates nuclear factor kappa B in cancer cells versus normal cells. Arch Biochem Biophys.

[CR3] Appari M, Babu KR, Kaczorowski A, Gross W, Herr I (2014). Sulforaphane, quercetin and catechins complement each other in elimination of advanced pancreatic cancer by miR-let-7 induction and K-ras inhibition. Int J Oncol.

[CR4] Bettuzzi S, Brausi M, Rizzi F, Castagnetti G, Peracchia G, Corti A (2006). Chemoprevention of human prostate cancer by oral administration of green tea catechins in volunteers with high-grade prostate intraepithelial neoplasia: a preliminary report from a one-year proof-of-principle study. Cancer Res.

[CR5] Boumahdi S, Driessens G, Lapouge G, Rorive S, Nassar D, Le Mercier M, Delatte B, Caauwe A, Lenglez S, Nkusi E, Brohée S, Salmon I, Dubois C, del Marmol V, Fuks F, Beck B, Blanpain C (2014). Sox2 controls tumour initiation and cancer stem-cell functions in squamous-cell carcinoma. Nature.

[CR6] Clarke MF, Dick JE, Dirks PB, Eaves CJ, Jamieson CH, Jones DL, Visvader J, Weissman IL, Wahl GM (2006). Cancer stem cells—perspectives on current status and future directions: AACR workshop on cancer stem cells. Cancer Res.

[CR7] Dalla Pozza E, Dando I, Biondani G, Brandi J, Costanzo C, Zoratti E, Fassan M, Boschi F, Melisi D, Cecconi D, Scupoli MT, Scarpa A, Palmieri M (2015). Pancreatic ductal adenocarcinoma cell lines display a plastic ability to bi-directionally convert into cancer stem cells. Int J Oncol.

[CR8] Driessens G, Beck B, Caauwe A, Simons BD, Blanpain C (2012). Defining the mode of tumour growth by clonal analysis. Nature.

[CR9] Duester G (2000). Families of retinoid dehydrogenases regulating vitamin A function: production of visual pigment and retinoic acid. Eur J Biochem.

[CR10] Fujiki H, Okuda T (1992). (−)-Epigallocatechin-3-gallate. Drugs Future.

[CR11] Fujiki H, Suganuma M (1993). Tumor promotion by inhibitors of protein phosphatases 1 and 2A: the okadaic acid class of compounds. Adv Cancer Res.

[CR12] Fujiki H, Suganuma M, Imai K, Nakachi K (2002). Green tea: cancer preventive beverage and/or drug. Cancer Lett.

[CR13] Fujiki H, Imai K, Nakachi K, Shimizu M, Moriwaki H, Suganuma M (2012). Challenging the effectiveness of green tea in primary and tertiary cancer prevention. J Cancer Res Clin Oncol.

[CR14] Fujiki H, Sueoka E, Watanabe T, Suganuma M (2015). Synergistic enhancement of anticancer effects on numerous human cancer cell lines treated with the combination of EGCG, other green tea catechins, and anticancer compounds. J Cancer Res Clin Oncol.

[CR15] Ginestier C, Hur MH, Charafe-Jauffret E, Monville F, Dutcher J, Brown M, Jacquemier J, Viens P, Kleer CG, Liu S, Schott A, Hayes D, Birnbaum D, Wicha MS, Dontu G (2007). ALDH1 is a marker of normal and malignant human mammary stem cells and a predictor of poor clinical outcome. Cell Stem Cell.

[CR16] Goodison S, Urquidi V, Tarin D (1999). CD44 cell adhesion molecules. Mol Pathol.

[CR17] Gu LT, Yang J, Su SZ, Liu WW, Shi ZG, Wang QR (2015). Green tea polyphenols protects cochlear hair cells from ototoxicity by inhibiting Notch signalling. Neurochem Res.

[CR18] Imai K, Suga K, Nakachi K (1997). Cancer-preventive effects of drinking green tea among a Japanese population. Prev Med.

[CR19] Jang GB, Kim JY, Cho SD, Park KS, Jung JY, Lee HY, Hong IS, Nam JS (2015). Blockade of Wnt/β-catenin signaling suppresses breast cancer metastasis by inhibiting CSC-like phenotype. Sci Rep.

[CR20] Jeter CR, Badeaux M, Choy G, Chandra D, Patrawala L, Liu C, Calhoun-Davis T, Zaehres H, Daley GQ, Tang DG (2009). Functional evidence that the self-renewal gene NANOG regulates human tumor development. Stem Cells.

[CR21] Jones PH, Watt FM (1993). Separation of human epidermal stem cells from transit amplifying cells on the basis of differences in integrin function and expression. Cell.

[CR22] Kashyap V, Rezende NC, Scotland KB, Shaffer SM, Persson JL, Gudas LJ, Mongan NP (2009). Regulation of stem cell pluripotency and differentiation involves a mutual regulatory circuit of the NANOG, OCT4, and SOX2 pluripotency transcription factors with polycomb repressive complexes and stem cell microRNAs. Stem Cells Dev.

[CR23] Kuzuhara T, Sei Y, Yamaguchi K, Suganuma M, Fujiki H (2006). DNA and RNA as new binding targets of green tea catechins. J Biol Chem.

[CR24] Lee SH, Nam HJ, Kang HJ, Kwon HW, Lim YC (2013). (−)-Epigallocatechin-3-gallate attenuates head and neck cancer stem cell traits through suppression of Notch pathway. Eur J Cancer.

[CR25] Li YJ, Wu SL, Lu SM, Chen F, Guo Y, Gan SM, Shi YL, Liu S, Li SL (2015). (−)-Epigallocatechin-3-gallate inhibits nasopharyngeal cancer stem cell self- renewal and migration and reverses the epithetial-mesenchymal transition via NF-κB p65 inactivation. Tumor Biol.

[CR26] Lin CH, Shen YA, Hung PH, Yu YB, Chen YJ (2012). Epigallocatechin gallate, polyphenol present in green tea, inhibits stem-like characteristics and epithelial- mesenchymal transition in nasopharyngeal cancer cell lines. BMC Complement Alern Med.

[CR27] Mineva ND, Paulson KE, Naber SP, Yee AS, Sonenshein GE (2013). Epigallocatechin- 3-gallate inhibits stem-like inflammatory breast cancer cells. PLoS One.

[CR28] Moyle CW, Cerezo AB, Winterbone MS, Hollands WJ, Alexeev Y, Needs PW, Kroon PA (2015). Potent inhibition of VEGFR-2 activation by tight binding of green tea epigallocatechin gallate and apple procyanidins to VEGF: relevance to angiogenesiss. Mol Nutr Food Res.

[CR29] Nakachi K, Suemasu K, Suga K, Takeo T, Imai K, Higashi Y (1998). Influence of drinking green tea on breast cancer malignancy among Japanese patients. Jpn J Cancer Res.

[CR30] Nakachi K, Matsuyama S, Miyake S, Suganuma M, Imai K (2000). Preventive effects of drinking green tea on cancer and cardiovascular disease: epidemiological evidence for multiple targeting prevention. BioFactor.

[CR31] Nishimura N, Hartomo TB, Pham TV, Lee MJ, Yamamoto T, Morikawa S, Hasegawa D, Takeda H, Kawasaki K, Kosaka Y, Yamamoto N, Kubokawa I, Mori T, Yanai T, Hayakawa A, Takeshima Y, Iijima K, Matsuo M, Nishio H (2012). Epigallocatechin gallate inhibits sphere formation of neuroblastoma BE(2)-C cells. Environ Health Prev Med.

[CR32] Oh S, Gwak J, Park S, Yang CS (2014). Green tea polyphenol EGCG suppresses Wnt/β-catenin signaling by promoting GSK-3β- and PP2A-independent β-catenin phosphorylation/degradation. BioFactor.

[CR33] Ohga N, Hida K, Hida Y, Muraki C, Tsuchiya K, Matsuda K, Ohiro Y, Totsuka Y, Shindoh M (2009). Inhibitory effects of epigallocatechin-3-gallate, a polyphenol in green tea, on tumor-associated endothelial cells and endothelial progenitor cells. Cancer Sci.

[CR34] Okabe S, Suganuma M, Hayashi M, Sueoka E, Komori A, Fujiki H (1997). Mechanisms of growth inhibition of human cancer cell line, PC-9, by tea polyphenols. Jpn J Cancer Res.

[CR36] Pan X, Zhao B, Song Z, Han S, Wang M (2016). Estrogen receptor-α36 is involved in epigallocatechin-3-gallate induced growth inhibition of ER-negative breast cancer stem/progenitor cells. J Pharmacol Sci.

[CR37] Pang Z, Kushiyama A, Sun J, Kikuchi T, Yamazaki H, Iwamoto Y, Koriyama H, Yoshida S, Shimamura M, Higuchi M, Kawano T, Takami Y, Rakugi H, Morishita R, Nakagami H (2017) Glial fibrillary acidic protein (GFAP) is a novel biomarker for the prediction of autoimmune diabetes. FASEB J pii:fj.201700110R. doi: 10.1096/fj.201700110R 10.1096/fj.201700110R 28546444

[CR38] Peinado H, Olmeda D, Cano A (2007). Snail, Zeb and bHLH factors in tumour progression: an alliance against the epithelial phenotype?. Nat Rev Cancer.

[CR39] Polyak K, Weinberg RA (2009). Transitions between epithelial and mesenchymal states: acquisition of malignant and stem cell traits. Nat Rev Cancer.

[CR40] Prince ME, Sivanandan R, Kaczorowski A, Wolf GT, Kaplan MJ, Dalerba P, Weissman IL, Clarke MF, Ailles LE (2007). Identification of a subpopulation of cells with cancer stem cell properties in head and neck squamous cell carcinoma. Proc Natl Acad Sci USA.

[CR41] Sarkar A, Hochedlinger K (2013). The sox family of transcription factors: versatile regulators of stem and progenitor cell fate. Cell Stem Cell.

[CR42] Shimizu M, Fukutomi Y, Ninomiya M, Nagura K, Kato T, Araki H, Suganuma M, Fujiki H, Moriwaki H (2008). Green tea extracts for the prevention of metachronous colorectal adenomas: a pilot study. Cancer Epidemiol Biomarkers Prev.

[CR43] Shin CM, Lee DH, Seo AY, Lee HJ, Kim SB, Son WC, Kim YK, Lee SJ, Park SH, Kim N, Park YS, Yoon H (2017). Green tea extracts for the prevention of metachronous colorectal polyps among patients who underwent endoscopic removal of colorectal adenomas: a randomized clinical trial. Clin Nutr pii.

[CR44] Sobrado VR, Moreno-Bueno G, Cubillo E, Holt LJ, Nieto MA, Portillo F, Cano A (2009). The class I bHLH factors E2-2A and E2-2B regulate EMT. J Cell Sci.

[CR555] Stearns ME, Wang M (2011). Synergistic effects of the green tea extract epigallocatechin-3-gallate and
taxane in eradication of malignant human prostate tumors. Transl Oncol.

[CR45] Stingl JC, Ettrich T, Muche R, Wiedom M, Brockmöller J, Seeringer A, Seufferlein T (2011). Protocol for minimizing the risk of metachronous adenomas of the colorectum with green tea extract (MIRACLE): a randomised controlled trial of green tea extract versus placebo for nutriprevention of metachronous colon adenomas in the elderly population. BMC Cancer.

[CR46] Suganuma M, Okabe S, Oniyama M, Tada Y, Ito H, Fujiki H (1998). Wide distribution of [^3^H](−)-epigallocatechin gallate, a cancer preventive tea polyphenol, in mouse tissue. Carcinogenesis.

[CR47] Suganuma M, Okabe S, Kai Y, Sueoka N, Sueoka E, Fujiki H (1999). Synergistic effects of (−)-epigallocatechin gallate with (−)-epicatechin, sulindac, or tamoxifen on cancer-preventive activity in the human lung cancer cell line PC-9. Cancer Res.

[CR48] Suganuma M, Kurusu M, Suzuki K, Tasaki E, Fujiki H (2006). Green tea polyphenol stimulates cancer preventive effects of celecoxib in human lung cancer cells by upregulation of *GADD153* gene. Int J Cancer.

[CR49] Suganuma M, Saha A, Fujiki H (2011). New cancer treatment strategy using combination of green tea catechins and anticancer drugs. Cancer Sci.

[CR50] Suganuma M, Takahashi A, Watanabe T, Iida K, Matsuzaki T, Yoshikawa HY, Fujiki H (2016). Biophysical approach to mechanisms of cancer prevention and treatment with green tea catechins. Molecules.

[CR51] Sugihara E, Saya H (2013). Complexity of cancer stem cells. Int J Cancer.

[CR52] Takahashi A, Watanabe T, Mondal A, Suzuki K, Kurusu-Kanno M, Li Z, Yamazaki T, Fujiki H, Suganuma M (2014). Mechanism-based inhibition of cancer metastasis with (−)-epigallocatechin gallate. Biochem Biophys Res Commun.

[CR53] Tang SN, Singh C, Nall D, Meeker D, Shankar S, Srivastava RK (2010). The dietary bioflavonoid quercetin synergizes with epigallocatechin gallate (EGCG) to inhibit prostate cancer stem cell characteristics, invasion, migration and epithelial- mesenchymal transition. J Mol Signal.

[CR54] Tang SN, Fu J, Nall D, Rodova M, Shankar S, Srivastava RK (2012). Inhibition of sonic hedgehog pathway and pluripotency maintaining factors regulate human pancreatic cancer stem cell characteristics. Int J Cancer.

[CR55] Taniguchi S, Fujiki H, Kobayashi H, Go H, Miyado K, Sadano H, Shimokawa R (1992). Effect of (−)-epigallocatechin gallate, the main constituent of green tea, on lung metastasis with mouse B16 melanoma cell lines. Cancer Lett.

[CR56] Thiery JP, Acloque H, Huang RY, Nieto MA (2009). Epithelial-mesenchymal transitions in development and disease. Cell.

[CR57] Toden S, Tran HM, Tovar-Camargo OA, Okugawa Y, Goel A (2016). Epigallocatechin- 3-gallate targets cancer stem-like cells and enhances 5-fluorouracil chemosensitivity in colorectal cancer. Oncotarget.

[CR58] Tsao AS, Liu D, Martin J, Tang XM, Lee JJ, El-Naggar AK, Wistuba I, Culotta KS, Mao L, Gillenwater A, Sagesaka YM, Hong WK, Papadimitrakopoulou V (2009). Phase II randomized, placebo-controlled trial of green tea extract in patients with high-risk oral premalignant lesions. Cancer Prev Res.

[CR59] Vayalil PK, Katiyar SK (2004). Treatment of epigallocatechin-3-gallate inhibits matrix metalloproteinases-2 and -9 via inhibition of activation of mitogen-activated protein kinases, c-jun and NF-kappa B in human prostate carcinoma DU-145 cells. Prostate.

[CR60] Wang X, Zheng M, Liu G, Xia W, McKeown-Longo PJ, Hung MC, Zhao J (2007). Krüppel-like factor 8 induces epithelial to mesenchymal transition and epithetial cell invasion. Cancer Res.

[CR61] Wang P, Henning SM, Heber D, Vadgama JV (2015). Sensitization to docetaxel in prostate cancer cells by green tea and quercetin. J Nutr Biochem.

[CR62] Watanabe T, Kuramochi H, Takahashi A, Imai K, Katsuta N, Nakayama T, Fujiki H, Suganuma M (2012). Higher cell stiffness indicating lower metastatic potential in B16 melanoma cell variants and in (−)-epigallocatechin gallate-treated cells. J Cancer Res Clin Oncol.

[CR63] Wubetu GY, Shimada M, Morine Y, Ikemoto T, Ishikawa D, Iwahashi S, Yamada S, Saito Y, Arakawa Y, Imura S (2016). Epigallocatechin gallate hinders human hepatoma and colon cancer sphere formation. J Gastroenterol Hepatol.

[CR64] Yang J, Weinberg RA (2008). Epithelial-mesenchymal transition: at the crossroads of development and tumor metastasis. Dev Cell.

[CR65] Yoshida GJ, Saya H (2016). Therapeutic strategies targeting cancer stem cells. Cancer Sci.

[CR66] Yoshizawa S, Horiuchi T, Suganuma M, Nishiwaki S, Yatsunami J, Okabe S, Okuda T, Muto Y, Frenkel K, Troll T, Fujiki H (1992). Penta-*O*-galloyl-β-d-glucose and (−)-epigallocatechin gallate. ACS Symp Ser.

[CR67] Yu SL, Chen HY, Chang GC, Chen CY, Chen HW, Singh S, Cheng CL, Yu CJ, Lee YC, Chen HS, Su TJ, Chiang CC, Li HN, Hong QS, Su HY, Chen CC, Chen WJ, Liu CC, Chan WK, Chen WJ, Li KC, Chen JJ, Yang PC (2008). MicroRNA signature predicts survival and relapse in lung cancer. Cancer Cell.

[CR68] Yu CC, Chen PN, Peng CY, Yu CH, Chou MY (2016). Suppression of miR-204 enables oral squamous cell carcinomas to promote cancer stemness, EMT traits, and lymph node metastasis. Oncotaget.

[CR69] Zhang XT, Kang LG, Ding L, Vranic S, Gatalica Z, Wang ZT (2011). A positive feedback loop of ER-α36/EGFR promotes malignant growth of ER-negative breast cancer cells. Oncogene.

[CR70] Zhang Y, Wang SX, Ma JW, Li HY, Ye JC, Xie SM, Du B, Zhong XY (2015). EGCG inhibits properties of glioma stem-like cells and synergizes with temozolomide through downregulation of P-glycoprotein inhibition. J Neurooncol.

[CR71] Zhu J, Jiang Y, Yang X, Wang S, Xie C, Li X, Li Y, Chen Y, Wang X, Meng Y, Zhu M, Wu R, Huang C, Ma X, Geng S, Wu J, Zhong C (2017). Wnt/β-catenin pathway mediates (−)-epigallocatechin-3-gallate (EGCG) inhibition of lung cancer stem cells. Biochemical Biophys Res Commun.

